# Patient-Reported Outcomes During Neoadjuvant Therapy for Gastrointestinal Cancer and Their Association with Postoperative Complications

**DOI:** 10.1007/s12029-025-01268-y

**Published:** 2025-07-02

**Authors:** Alexander H. Shannon, Samantha M. Ruff, Marilly Palettas, Angela Sarna, Emily Huang, Peter J. Kneuertz, Mary Dillhoff, Aslam Ejaz, Timothy M. Pawlik, Jordan M. Cloyd

**Affiliations:** 1 Division of Surgical Oncology, The Ohio State University Wexner Medical Center, 410 W 10Th Ave, N-907 Doan Hall, Columbus, OH 43210 USA; 2 University of Illinois-Chicago Health System, Chicago, IL USA

**Keywords:** Patient-reported outcome measures, Preoperative therapy Pancreatic cancer, Colorectal cancer, Hepatopancreatobiliary surgery, Esophageal cancer

## Abstract

**Purpose:**

Neoadjuvant therapy (NT) given before surgery for gastrointestinal (GI) malignancies can lead to adverse events. Whether patient-reported outcomes (PRO) or quality of life (QOL) during NT is predictive of postoperative complications is unknown.

**Methods:**

A planned secondary analysis of patients with GI cancers undergoing NT utilized a customized mobile-phone application (app) to measure real-time PROs and monthly QOL using FACT-G (Functional Assessment of Cancer Therapy-General). Among surgical patients, the association between QOL and PROs and postoperative Clavien-Dindo grade ≥ 2 complications was analyzed using univariate analyses.

**Results:**

Among 104 patients enrolled, 69 (66%) underwent surgery following NT and 20 (28.9%) experienced 30-day complications. There were no differences in baseline demographics, NT duration, or cancer type between Complications and No Complications groups (all *p* > 0.05). QOL scores at NT start (mean FACT-G Complications 76.1 vs No Complications 75.2), and changes in QOL during NT did not differ between the two cohorts (*p* > 0.05). PRO entries of those who experienced complications were more likely to report lack of appetite (25.9% vs 14.2%; *p* < 0.001) and pain (36.6% vs 18.7%; *p* < 0.001) but less likely to report fatigue (31.9% vs 41.6%; *p* = 0.009), anxiety (18.1% vs 39.1%; *p* < 0.001), trouble sleeping (20.8% vs 39.1%; *p* < 0.001), lack of focus (5.6% vs 18.5%; *p* < 0.001), depression (0.5% vs 14%; *p* < 0.001), and frustration (13.9% vs 21.4%; *p* = 0.01).

**Conclusion:**

In this prospective cohort study, specific PROs were associated with postoperative complications among those who underwent surgical resection. Further research is needed to assess whether preoperative PROs can guide patient-centered interventions mitigating postoperative complications.

## Introduction

The delivery of non-surgical cancer treatments such as chemotherapy or radiotherapy prior to surgical resection, known as neoadjuvant therapy (NT), has increased in recent years, and it represents the standard of care for many specific gastrointestinal (GI) cancers such as rectal, esophageal, and gastric [1–4]. Benefits of NT include downstaging locally advanced cancers, improving locoregional control, organ preservation, and allowing for minimally invasive surgical techniques [5–7]. Additionally, NT provides valuable information regarding tumor biology and the effectiveness of therapies. Finally, NT improves receipt of multimodality therapy prior to surgical resection, which can be morbid and delay subsequent adjuvant therapy [8, 9].

However, as NT becomes increasingly standardized in the management of GI malignancies, its impact on postoperative complications needs to be expounded. NT can cause significant symptoms in patients, has a high incidence of adverse events, and impacts both emotional and physical well-being [10, 11]. In some cases, aggressive preoperative therapy can even lead to sufficient performance status decline to render patients no longer eligible for surgery [12]. In a prior prospective study of patients undergoing NT for GI malignancies, we utilized a customized smartphone application to measure quality of life (QOL) and patient reported outcomes (PROs) during NT and found that symptom burden was high including fatigue, insomnia, and anxiety [13]. Whether changes in PROs or QOL during NT is predictive of postoperative complications has yet to be determined.

Surgery for GI and hepatopancreaticobiliary (HPB) cancer is complex and can be associated with major morbidity for patients [14]. Complications may increase likelihood for failure to rescue, impacts QOL, and/or delay crucial adjuvant therapy for patients. Thus, early identification of patients at high risk for postoperative complications may enable modifications of treatments and patient centered interventions to mitigate these complications. Therefore, the objective of this planned secondary analysis of prospectively collected clinical and PROs data was to determine if PROs or QOL during NT were predictive of 30-day postoperative complications following surgery for GI and HPB malignancies.

## Methods

### Patient Population

Patients with GI or HPB cancer, including pancreatic, liver or biliary, colorectal, esophageal, or gastric, undergoing NT were enrolled in the study from August 2020 to December 2021. Patients were included if they were older than 18, had known GI or HPB cancer, had not yet started NT, owned a smart phone, and were English speaking. QOL scores and PROs were prospectively recorded during NT. The preliminary results and study protocol of this trial have been previously published [13]. A mobile smartphone application (app) called SeamlessMD was customized to allow patients to record mood and symptoms in real-time, as well as administer assessments of QOL monthly, and free text journaling during NT.

### Clinical Outcomes

QOL was assessed using a validated 27-item questionnaire called the Functional Assessment of Cancer Therapy-General (FACT-G) survey, which evaluates QOL based off four subcategories: physical well-being (PWB), social well-being (SWB), emotional well-being (EWB), and functional well-being (FWB). Symptoms assessed were nausea/vomiting, diarrhea, constipation, no appetite, pain, fatigue, anxiety, trouble sleeping, lack of focus, depression, hopelessness, and frustration and were recorded as present or not. Mood was recorded on 0–4 scale with lower and higher scores reflecting “poor mood” and “good mood,” respectively. Additionally, demographic data, including cancer type and type of surgery, as well as postoperative complications Clavien-Dindo grade 2 or above, were collected on all patients. The Clavien-Dindo classification is used to grade the severity of postoperative complications and has five grades: Grade I, any deviation from normal postoperative course without need for pharmacologic or procedural interventions; Grade II, complications requiring pharmacologic treatment with drugs; Grade III, complications requiring surgical, endoscopic, or radiological intervention; Grade IV, life-threatening complications requiring intensive care unit management; Grade V, death of a patient [15].

### Data Analysis

The cohort of patients who underwent surgery was divided into two groups based on the occurrence of 30-day postoperative complications Clavien-Dindo grade 2 or above (Complications and No Complications, respectively). Demographic and clinical data were compared between the two groups using descriptive statistics with a *p*-value < 0.05 to denote significance. Univariate analysis was used to determine associations between demographic and clinical factors and 30-day postoperative complications. The study was approved by the clinical scientific review committee and institutional review board (#2020C0071).

## Results

### Patient Characteristics

Of the 104 patients enrolled in the prospective cohort, 69 (66%) underwent surgery and were included in the study. The mean age of the cohort was 59.0 ± 11.8 years, 47.8% were male, and 92.8% were Caucasian. Types of cancer included were colorectal (*n* = 36, 52.2%), esophageal (*n* = 9, 13%), gastric (*n* = 4, 5.8%), and pancreatic (*n* = 20, 29%).

Among the 69 patients who underwent surgery, 20 (29%) had a 30-day complication Clavien-Dindo ≥ 2 whereas 49 (71%) did not. Complications were graded as Clavien-Dindo grade 3 (*n* = 10, 50%), grade 2 (*n* = 8, 40%), and grade 4 (*n* = 2, 10%); there were no 30-day mortalities in either group. Overall, there was no difference in baseline demographics including age, gender, race, marital status, number of co-morbidities, alcohol use, distance traveled to NT, and duration of NT between patients who did and did not have complications (all *p* > 0.05, Table 1). While there was a significant difference in length of hospital stay between the two groups (Complications 9.6 ± 5.7 days vs No Complications 6.2 ± 3.6 days, *p* = 0.002), there was no association between 30-day complications and type of cancer or type of surgery performed (*p* = 0.28 and *p* = 0.90, respectively).

**Table 1:** Clinical and demographic factors between Complications and No Complications groups

Characteristic	Complications (*N* = 20)^1^	No Complications (*N* = 49)^1^	*p*-value^2^
**Age**	63 (11)	57 (12)	0.09
**Gender**			0.45
Female	9 (45%)	27 (55%)	
Male	11 (55%)	22 (45%)	
**Race**			0.8
African American	1 (5%)	3 (6%)	
Caucasian	19 (95%)	45 (92%)	
Other	0 (0%)	1 (2%)	
**Marital status**			0.56
Divorced	1 (5%)	2 (4%)	
Married/partner	12 (60%)	31 (63%)	
Single	3 (15%)	12 (24%)	
Widowed	3 (15%)	2 (4%)	
Other	1 (5%)	2 (4%)	
**Miles traveled to NT**	59 (39)	55 (36)	0.73
**Comorbidities**			0.25
0	11 (55%)	34 (69%)	
1 or more	9 (45%)	15 (31%)	
**Smoker**			0.23
Current	4 (20%)	6 (12%)	
Former	10 (50%)	17 (35%)	
Never	6 (30%)	26 (51%)	
**Current alcohol use**			0.41
No	13 (65%)	26 (53%)	
Yes	7 (35%)	23 (45%)	
**Cancer type**			0.29
Colorectal	10 (50%)	26 (53%)	
Esophagus	3 (15%)	6 (12%)	
Gastric	3 (15%)	1 (2%)	
Pancreas	4 (20%)	16 (33%)	
**Neoadjuvant treatment at OSU**	14 (70%)	32 (65%)	0.71
**Duration neoadjuvant treatment (months)**	2.35 (1.65)	2.96 (1.82)	0.29
**Hospital length of stay (days)**	9.6 (5.7)	6.2 (3.6)	0.002

Abbreviations: *NT* neoadjuvant therapy, *OSU* Ohio State University

^1^*n* (%); Mean (SD)

^2^Pearson’s chi-squared test; Wilcoxon rank sum test; Fisher’s exact test

### Patient Reported Outcomes

During the study, the Complications and No Complications groups completed 216 and 844 symptom tracker entries using the mobile application, respectively. Overall, 18 (90%) Complications patients and 43 (87.8%) No Complications patients had at least one entry during NT (*p* = 0.79), and the mean number of entries per person were 11.4 and 19.6 (*p* = 0.35), respectively.

For the Complications group, the most reported symptoms during NT were pain (36.6%), fatigue (31.9%), and lack of appetite (25.9%) whereas the least reported symptoms during NT were lack of focus (5.6%), depression (0.46%), and hopelessness (0%). For the No Complications group, the most reported symptoms during NT were fatigue (41.6%), anxiety (39.1%), and trouble sleeping (39.1%) whereas the least reported symptoms during NT were lack of focus (18.5%), depression (14%), and hopelessness (5.1%).

### Association with Complications

**Fig. 1:**
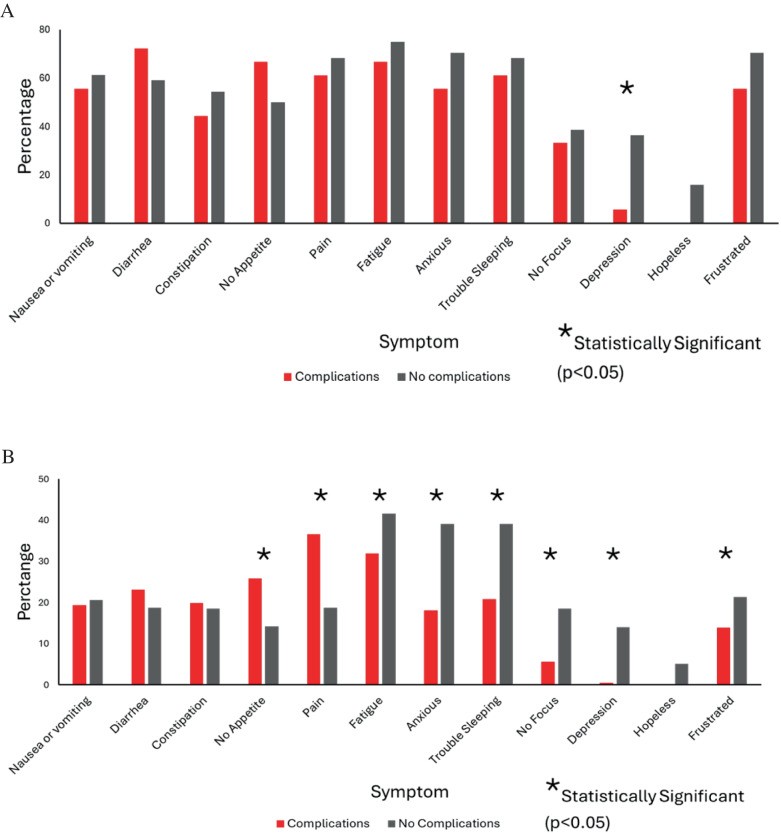
**A** Percentage of patients reporting symptoms at least once during NT. **B** Percentage of entries reporting symptoms during NT. Abbreviations: NT, neoadjuvant therapy

On univariate analysis, when assessing the proportion of patients who reported a symptom at least once during NT, there were no differences between groups in all symptoms, except for depression which was significantly higher in the No Complications group (*p* = 0.02, Fig. 1A). However, significant differences were seen between the two groups when comparing the proportion of all PRO entries reported positive, including lack of appetite (Complications 25.9% vs No Complications 14.2%; *p* < 0.001), pain (36.6% vs 18.7%; *p* < 0.001), fatigue (31.9% vs 41.6%; *p* = 0.009), anxiety (18.1% vs 39.1%; *p* < 0.001), trouble sleeping (20.8% vs 39.1%; *p* < 0.001), lack of focus (5.6% vs 18.5%; *p* < 0.001), depression (0.5% vs 14%; *p* < 0.001), and frustration (13.9% vs 21.4%; *p* = 0.01) (Fig. 1B).

**Fig. 2:**
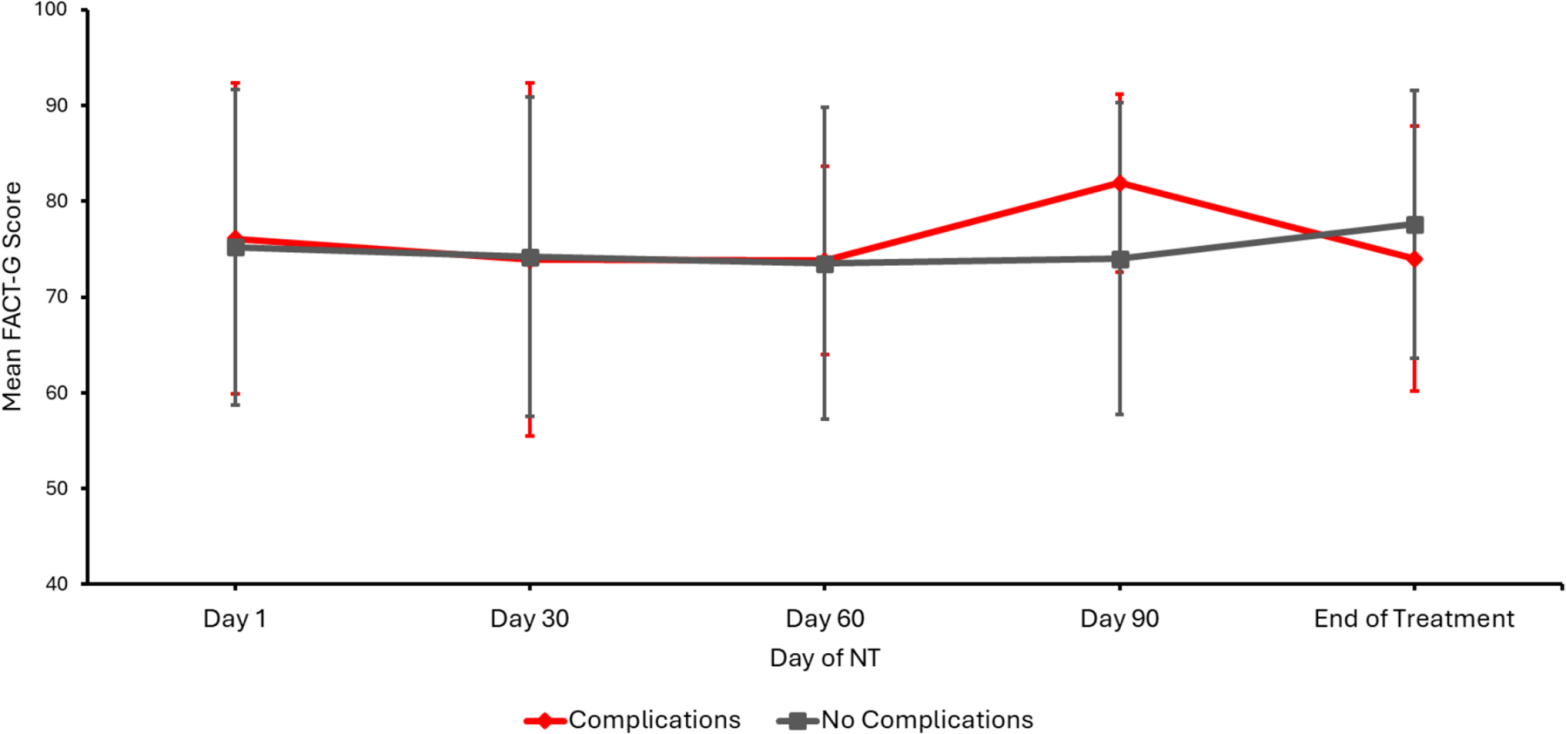
Prospective quality of life assessment between Complications and No Complications groups during NT. Abbreviations: NT, neoadjuvant therapy; FACT-G, Functional Assessment of Cancer Therapy-General

Among the 216 mood entries on a scale of 0–4 (0 indicating poor mood and 4 indicating good mood), there was no difference between the two cohorts in average mood score (1.71 vs 1.75). There was no significant difference between the two groups in baseline QOL scores at the start of NT (mean day 0 FACT-G score 76.1 vs 75.2) nor were there differences in the change of FACT-G scores over the duration of NT (*p* > 0.05, Fig. 2).

## Discussion

In this secondary analysis of a prospective cohort study assessing real-time PROs and QOL during NT for GI and HPB cancers, certain PROs during NT including lack of appetite and pain were associated with increased 30-day postoperative complications, whereas changes in longitudinal QOL were not. These data warrant further evaluation of the prognostic significance of PROs during NT. Given its increasing use prior to surgery for many GI and HPB malignancies, identifying patients at higher risk for postoperative complications may allow providers to alter treatment plans and/or provide patient-centered interventions to mitigate the increased risk of complications.

Multimodal therapy is a cornerstone principle for most GI cancers. While traditionally, adjuvant therapy was delivered after surgical resection, NT prior to surgery has become the standard of care in many stages of gastric, esophageal, pancreatic, and rectal cancers [2, 4, 16–18]. Interestingly, the majority of literature has found that the receipt of NT does not negatively influence the incidence of postoperative complications compared with upfront surgery [19–23]. Indeed, in some situations, NT may be protective of postoperative complications (e.g. neoadjuvant radiation on the incidence of postoperative pancreatic fistula following pancreatectomy) [24]. In addition, the neoadjuvant period has often been touted as an opportunity to optimize patients with borderline functional status through formal prehabilitation [25, 26]. Furthermore, NT may be associated with improved postoperative outcomes by selecting those patients with better tumor biology and functional status prior to undergoing major abdominal surgery [27, 28]. Nevertheless, chemotherapy and radiation therapy used for GI cancers, in general, can lead to significant toxicity, and whether PROs during NT is predictive of postoperative outcomes among those who do undergo surgery has not been previously investigated.

There has been little study assessing the role of preoperative PROs in predicting postoperative complications in patients undergoing major cancer surgery. Much of the existing literature explores perioperative or postoperative PROs and their effect on complications. A secondary analysis of the COST trial, which assessed laparoscopic vs open resection for colon cancer, showed that poor perioperative QOL scores were an early indicator for patients at risk for postoperative complications [29]. Yang et al. [30] found that postoperative PROs such as coughing, daily activity, and pain in patients undergoing thoracic surgery for lung tumors were associated with post-discharge complications. On the other hand, it is well known that preoperative baseline frailty measures correlate with risk of postoperative complications [31, 32]. Indeed, one study assessing preoperative patient-reported outcomes measurement information system (PROMIS) physical function showed that moderate to severe physical function scores were predictive of postoperative complications in patients undergoing elective colorectal surgery [33]. At the same time, research has found that PROs are predictors of mortality and survival in colon cancer [34, 35].

The utilization of PROs in measuring outcomes for cancer patients receiving therapy is crucial to provide quality patient care, and there is a growing body of literature to underscore its importance. PROs allow providers to gauge patient’s well-being and tolerance of therapy as well as tumor response [36]. Real-time monitoring of PROs allows providers to intervene on patients at risk for poor outcomes, thereby increasing patient’s satisfaction with care, improving QOL scores, and enhancing patient outcomes during treatment [37–40]. Indeed, a systematic review found that measuring PROs was associated with increased patient satisfaction as well as improved symptom control during their cancer care [40]. The advent of novel technologies such as mobile phone applications or tablets has opened new doors for providers to help monitor PRO and offer opportunities for patients to be more engaged and in tune with their care. Basch et al. [37] showed that utilization of tablets allowing patients to record symptoms during cancer therapy for solid tumors had high quality of life scores, few emergency room visits, and were more likely to complete NT. Additionally, Rossi et al. [41] showed that PROs gained through remote patient telemonitoring showed potential for predicting postoperative complications. Further research into the use of novel technologies to monitor PROs is needed to intervene on patients at high risk for postoperative complications.

Our results further highlight the importance of PROs in providing the best quality cancer care for patients. While longitudinal QOL scores during NT were not predictive of 30-day complications, multiple PROs such as lack of appetite and pain during NT were associated with postoperative complications whereas others such as fatigue, anxiety, depression, and frustration were associated with not having a complication. Early identification of patients with worsening PROs during NT may allow for targeted interventions aimed at optimizing patients to complete therapy and prepare for surgery. For instance, in patients with lack of appetite, evaluating nutritional status and providing appetite stimulants may help to optimize patients prior to surgery. Additionally, multimodality pain regiments to treat pain during NT may improve QOL scores and functional status preoperatively thus potentially improving postoperative outcomes. Interestingly, certain PROs, including fatigue, anxiety, trouble sleeping, lack of focus, depression, and frustration, were inversely associated with 30-day complications. These findings are difficult to explain considering research that shows preoperative emotional well-being is associated with postoperative outcomes [42, 43]. It is possible that patients who more frequently engaged with the app and self-reported emotional symptoms were better surgical candidates. Another possibility is that the self-reporting emotional symptoms was therapeutic and allowed for optimization of their emotional well-being prior to surgery. Indeed, optimizing one’s mental status prior to surgery has shown to improve postoperative QOL [44]. NT creates a significant emotional and physical burden on patients, and this emphasizes the importance of PRO in identifying patients who may be at risk for postoperative complications [19, 45].

While interesting, several limitations of the study should be acknowledged. First, the patient population was heterogeneous in terms of underlying pathology with several different malignancies included, each of which have different prognoses and NT regimens. For example, watch and wait protocols for patients with stage 2 or 3 rectal cancers have been adopted in many institutions, thus decreasing the number of patient undergoing surgical resection in our cohort [3]. Secondly, our cohort was limited to English speaking adults with access to a smartphone at a single institution. This may limit the generalizability of our findings especially to those of lower socioeconomic status, non-English speakers, or limited access to technology. Finally, while this represented a pre-planned secondary analysis of a prospective cohort study, the final sample size was relatively small and limited our ability to conduct multivariable analyses to control for confounders. Based on these limitations, these findings should be considered hypothesis-generating, and additional research is needed to evaluate the role of PRO monitoring during NT.

In conclusion, among patients undergoing NT for GI/HPB malignancies, several PROs, including lack of appetite and pain but not global QOL, were associated with 30-day postoperative complications. Further research is needed as to whether preoperative PROs can guide the need for patient-centered interventions aimed at mitigating postoperative complications following major cancer surgery.

## Data Availability

No datasets were generated or analysed during the current study.
